# The impact of Internet use on older adults’ attitudes toward positive aging: evidence from China

**DOI:** 10.3389/fpubh.2025.1624889

**Published:** 2026-01-07

**Authors:** Yalin Li, Ping Luo, Min Deng, Luyan Li, Qin Yin

**Affiliations:** School of Humanities and Management, Kunming Medical University, Kunming, Yunnan, China

**Keywords:** older people, the Internet, subjective age, empowerment, positive aging

## Abstract

**Objective:**

The concurrent trends of population aging and digitalization underscore the growing relevance of the Internet to older adults’ lifestyles and health. This paper explores the influence of Internet use on positive aging attitudes and its underlying mechanisms, with a specific analysis of the effects of different online functions. The findings are intended to inform efforts toward the digital empowerment of the older adults.

**Methods:**

Based on the 2018 survey data from the China Longitudinal Aging Social Survey (CLASS), a linear regression model is adopted for empirical analysis. Instrumental variable method and the Propensity-Score-Matching method is used in this study to conduct a robustness test on the impact results of Internet use. The Process3.0 plugin of SPSS was used to verify the mediating effects of life satisfaction and health comparison with peers on the relationship between Internet use and the age identity of older people.

**Results:**

It was found that Internet use has a significant positive impact on the rejuvenation of age identity in older adults. This relationship is partially mediated by both life satisfaction and health comparisons with peers, with the latter exhibiting a stronger mediating effect. In terms of online functions, those related to life services and social interaction exerted a significantly greater influence than entertainment and information functions.

**Conclusion:**

Based on the empowerment theory, it is recommended that policies first focus on improving Internet accessibility for older adults. Subsequently, smart older care services should be tailored to their needs, while initiatives encouraging intergenerational digital feedback and offline interaction should be strengthened.

## 1 Introduction

By the end of 2024, the population aged 65 and above in China was 220.23 million, accounting for 15.6% of the total national population (1). The gradual increase in the proportion of the older adults population is the basic form of China’s society in the future. In 2002, the World Health Organization released “Active Aging: A Policy Framework,” it advocating health, participation, and security as the pillars to establish a supportive and older adults-friendly society for the health and wellbeing of the older adults, and encouraging the improvement of the personal and social conditions for the older adults to participate in society, increasing the opportunities for the older population to maintain their independence (2). Continuous learning and self-improvement are the keys for the older adults to adapt to the development of the times and achieve health and longevity (3). Population aging and the digitalization of social life are advancing in parallel and many studies have shown that the use of the Internet has a positive impact on the physical and mental health of the older adults by increasing their social support, making it easier for them to access information, improving their health literacy and providing them with a rich recreational experience. The “Statistical Report on the Development of the Internet in China” shows that as of June 2025, the number of Internet users in China reached 1.123 billion, and the Internet penetration rate reached 79.7%. Among them, the Internet users aged 60 and above accounted for 52%, but the number of non-Internet users in this group accounted for 52.1% of the overall number of non-Internet users (4). The adaptation of digital technologies to the needs of the older adults and the provision of information accessibility services still need to be continuously promoted.

Research shows that the impact of the digital divide on the older adults is reflected in aspects such as objective social integration and subjective feelings (5). In the research on the mental health of the older adults, age identity reflects the individual’s subjective perception of the aging process. A younger subjective age correlates with a more positive aging experience. A younger age identity is associated with better physical and mental health as well as greater overall wellbeing among the older adults. It is a microscopic indicator of active aging and healthy aging (6), and an important manifestation of “the older adults live a happy life in their old age.” In related research, the impact of Internet use on the life satisfaction of the older adults is often seen, while research on the impact of Internet use on the positive aging attitude based on age identity is rarely seen. In addition, in the process of bridging the digital divide, the relationship between the elder’s preference for Internet functions and their mental state when using Internet content related to socializing, learning, living and entertainment is also worth analyzing and discussing. Based on the survey data released by the China Longitudinal Aging Social Survey, this paper explores the impact and mechanism of Internet use on the positive aging attitude of the older adults, and further analyzes the impact effects of various Internet functions, hoping to provide an empirical basis for the direction of digital empowerment for the older adults.

## 2 Literature review and research hypothesis

Age identity refers to an individual’s subjective judgment of their own age, which reflects the individual’s self-assessment of their position in the course of their life. It is commonly measured by the degree of perceived “oldness” or “youthfulness” compared to the actual age (7). People whose subjective age is younger than their actual age tend to have a more positive attitude toward aging, while those whose subjective age is older than their actual age feel older themselves and have a more negative attitude toward aging (8). Age identity is a more important predictive variable for physical and psychological wellbeing than the actual age (9). The tendency toward a younger subjective age is an important indicator of successful aging (10). Identifying factors that promote a youthful age identity among older adults has become a critical area of inquiry in health sociology.

Research has found that the age identity of the older adults is closely related to their actual age and health status. The older people with better health conditions and memory compared with their peers have a younger subjective age (11). In addition, the mental health and subjective wellbeing of the older adults are also related to age identity. The more the elder’s age identity tends to be younger, it can be predicted that the less depressive and anxious emotions the older adults experience, and the higher the level of life satisfaction (12). In the context of digital technology’s pervasive role in daily life, Internet use has emerged as a critical avenue for exploring the factors that shape age identity.

From a theoretical perspective, Internet use may influence older adults’ age identity by facilitating their “resocialization” process. According to Parsons’ socialization theory, major late-life events such as retirement, widowhood, and disability compel older adults to undergo re-socialization, as their initial socialization models become inadequate (13). In this process, the “digital inclusion” of the Internet is regarded as a key pathway to achieving “social inclusion.” Specifically, the use of the Internet can improve the level of family support and social support for the older adults, help build new social relationships and reduce the loneliness of the older adults, thereby enhancing their subjective wellbeing (14). Internet use enables older adults to transcend physical barriers, maintain and expand social networks, and reconstruct social roles, thereby mitigating the sense of aging resulting from social disconnection. On the other hand, social cognitive theory suggests that individual behavior is influenced by a combination of cognitive, environmental, and personal factors (15). In the digital inclusion of older adults, digital literacy is a key factor influencing their usage behavior. Some older adults, often due to lower learning capacity, confine their digital activities to simple leisure and social communication, demonstrating significantly weaker digital literacy in more complex scenarios such as transportation or online shopping (16). Moreover, the older adults often encounter difficulties in distinguishing the authenticity of online information and are also in a disadvantaged position at the cognitive level (17), making the impact of Internet use as a source of information does not have a significant impact on the physical and mental health of the older adults (18). When extensive free time is devoted to digital devices, some older adults may experience a passive form of engagement characterized by “short video retirement” and information cocoons. This pattern of use, reinforced by algorithmic personalization, can lead to excessive dependency on digital environments (19). Excessive Internet use can displace sleep time for the older adults and worsen their health conditions (20). The increase in Internet use will lead to a decrease in communication between respondents and family members, narrowing the scale of their social interactions, and instead increasing their depressive and lonely emotions (21). Within the older adult population, those who used the Internet frequently were more depressed than other older adults who did not use the Internet frequently (22). Therefore, the “high-frequency use” of smartphones by the older adults does not naturally translate into “high-empowerment use” (23). If used improperly, it may lead to complex ethical issues, which could run counter to the original intention of “active aging” (24). In summary, while a growing body of research has examined the impact and mechanisms of Internet use on older adults’ health, the findings are inconclusive. Furthermore, little attention has been paid to its specific effect on a positive aging mindset. Based on the above research and findings, we propose the first research hypothesis. H1: Using the Internet will significantly enhance the younger age identity of the older adults in China. That is, for all the older adults, those who use the Internet have a younger subjective age compared to those who do not use the Internet.

Beyond other pathways like continuous education, mass media serves as an instrumental channel for information dissemination, facilitating the resocialization of older adults (13). The older adults can conduct health management by browsing health portals and consulting, thereby improving their health level (25); the Internet can also strengthen the awareness of learning among the older adults, thus improving their physical and mental health levels (26); the Internet can promote the improvement of their cognitive functions and empower the older adults by influencing their interpersonal interactions (27). While increasing older adults’ self-efficacy (28), Internet use can also improve their loneliness (29), strengthen its youthful age identity. In 2015, the World Health Organization emphasized in its definition of healthy aging that “healthy aging is the process of developing and maintaining the functional ability that enables wellbeing in older age” (30). It emphasizes not the external “giving” of strength, but rather the stimulation of an individual’s or group’s inner potential through external support (such as resources, skills, opportunities). Against the backdrop of digital technology serving as a new production factor and power vector, the World Health Organization (WHO) released the “Global Strategy on Digital Health (2020–2025),” advocating for the development and utilization of digital technologies to enhance and manage health (31). Research suggests that healthy aging in the digital era is defined as the process of developing and maintaining the functional capacities necessary for a healthy life, a process powered by technological empowerment through modern information and digital tools (32). By providing health information, learning resources, and life conveniences, the Internet acts as an empowering tool that enhances self-management capabilities and efficacy among older adults. This empowerment stimulates intrinsic motivation and resource mobilization, with the resulting sense of control being central to a positive aging mindset. Therefore, digital empowerment should affect the younger age identity of the older adults through the intermediary of their self-assessment of physical and mental health. Based on the above analysis, this article puts forward the following research hypotheses: H2: The Internet affects the younger age identity of the older adults through the intermediary of their physical and mental pleasure. Specifically: H2a: Using the Internet is conducive to enhancing the older adults satisfaction with life, and thus maintaining a younger age identity. That is, life satisfaction plays a mediating role between Internet use and the younger subjective age. H2b: Using the Internet is conducive to improving the older adults health comparison scores with their peers, and thus maintaining a younger age identity. That is, the relative sense of health compared with their peers plays a mediating role between Internet use and the younger subjective age.

In summary, the internet’s influence on older adults’ age identity is complex and unlikely to be uniformly positive or negative, but rather contingent on specific usage patterns. Given this complexity, it is imperative to move beyond a simple use/non-use binary and develop a more nuanced understanding of these differential effects. This leads to the third hypothesis: H3a: Compared to recreational use, active Internet use for information and connection is predicted to foster a younger age identity by strengthening life satisfaction of the older people. H3b: In contrast, passive, recreational use is hypothesized to negatively predict a younger age identity, by increasing the risk of excessive use and encroaching on offline social time, thereby reducing life satisfaction.

## 3 Data sources and variable descriptions

### 3.1 Data sources

This paper uses the survey data released in 2018 by the China Longitudinal Aging Social Survey (CLASS) for statistical analysis. The survey adopts a stratified multi-stage probability sampling method, covering a total of 476 villages/neighborhood committees in 30 provinces/autonomous regions/municipalities directly under the Central Government across the country. The content involves rich information on the economic and social backgrounds and health conditions of the older adults, which meets the analysis needs of this paper. The latest survey obtained a total of 11,419 samples of the older adults. This paper selects samples of the people over 60 and under 100 for analysis. After deleting the missing values of variables related to the analysis, a total of 7,665 valid samples are finally obtained for statistical analysis.

### 3.2 Variable descriptions

The dependent variable of this study is age identity, which is operationalized as the difference between the actual age and the subjective age, and it is a continuous variable. The subjective age is measured by asking the question “E3-2: Most of the time, how old do you think you are?” The actual age is measured by asking the question “A2: What year were you born?” A positive value indicates that the older adults feel younger than their actual age, a negative value indicates that the older adults feel older than their actual age, and a value of 0 indicates that there is no obvious bias in the elder’s age identity.

This study focuses on the impact of the Internet usage behavior of the older adults on their age identity. Therefore, the independent variable is the Internet usage behavior, which is operationalized as a dichotomous variable. The frequency of Internet use is measured by asking the question “D21: Do you go online?” The response option “Never use the Internet” in the questionnaire is assigned a value of 0, and the options “Use the Internet several times a year,” “Use the Internet at least once a month,” “Use the Internet at least once a week,” and “Use the Internet every day” are combined and assigned a value of 1. In addition, this study also focuses on the impact of the elder’s use of the Internet for different purposes on their age identity. Therefore, the purposes of Internet usage are classified. Based on the question “D24-1: What do you usually do online?” The response options “Voice and video chat” and “Text chat” are combined into “Social Interaction,” “Read the news,” and “Browse articles/information” are combined into “Obtain Information,” “Watch videos,” and “Play games” are combined into “Entertainment,” and “Shopping,” “Transportation,” “Health Management,” “Investment and Financial Management,” and “Learning and Training” are combined into “Life Services.”

This paper also examines the influence mechanism of Internet use on age identity, and takes life satisfaction and relative self-assessment of health (compared with peers) as mediating variables. The life satisfaction is measured by asking the question “B17: Overall, are you satisfied with your current life?” And relative self-assessment of health is measured based on the question “B2: Compared to people your age, how would you rate your health condition?” The response options for both of the above questions are 5-point Likert scales. Combining existing research and relevant theoretical foundations, control variables are determined from the aspects of demographic characteristics, socioeconomic characteristics, regional characteristics, and physical health conditions. The specific variable information is shown in Table 1.

**Table 1:** Descriptive statistics of variables.

Variable name		Variable definition	Proportion (%)/mean value
Dependent variable	Age identity	Actual age—subjective age (continuous variable)	3.535
		Subjective age < actual age	61.94
		Subjective age = actual age	15.90
		Subjective age > actual age	22.15
Independent variable	Use Internet	Never use the Internet (reference group)	78.86
		Use the Internet several times a year/at least once a month/at least once a week/every day	21.14
	Internet social interaction	Do not use (reference group = 0)	80.91
		Use = 1	19.09
	Internet entertainment	Do not use (reference group = 0)	88.52
		Use = 1	11.48
	Internet information acquisition	Do not use (reference group = 0)	86.05
		Use = 1	13.95
	Internet life service	Do not use (reference group = 0)	95.11
		Use = 1	4.89
Mediating variable	Life satisfaction	Extremely dissatisfied = 0; somewhat dissatisfied = 1; average = 2; somewhat satisfied = 3; very satisfied = 4	2.777
	Self-assessed relative health	Much worse = 0; somewhat worse = 1; about the same = 2; somewhat better = 3; much better = 4	2.143
Control variable	Age	Samples of the older adults younger than 60 years old and older than 100 years old are removed	71.208
	Gender	Male (reference group = 0)	50.82
		Female = 1	49.18
	Ethnicity	Ethnic minority (reference group = 0)	5.34
		Han people = 1	94.66
	Marital status	Divorced, unmarried, widowed (reference group = 0)	29.95
		Married with a spouse	70.05
	Educational attainment	Illiterate (reference group = 0)	24.12
		Literate = 1	75.88
	Living arrangement	Not living alone (reference group = 0)	88.31
		Living alone = 1	11.69
	Type of residence	Urban (reference group = 0)	59.99
		Rural = 1	40.01
	Number of surviving children	Number of surviving sons + number of surviving daughters	2.54
	Employment status	Not engaged in remunerated work/activity (reference group = 0)	74.82
		Engaged in remunerated work/activity = 1	25.18
	Status of obtaining government endowment insurance (allowance)	Without various social endowment Insurances, subsidies, and minimum living security (reference group = 0)	7.81
		With various social endowment insurances/subsidies/minimum living security = 1	92.19
	Personal income	Take the logarithm	8.179
	Housing ownership status	Without housing (reference group = 0)	4.77
		Owning one or more houses = 1	95.23
	ADL	Needing assistance in one of the following activities: dressing/bathing/eating/toileting/incontinence control/indoor walking = 1	11.00
		Needing no assistance in the above activities (reference group = 0)	89.00
	IADL disability status	Needing assistance in one of the following activities: going up and down stairs/walking outdoors/taking public transport/shopping/cooking/doing housework/managing finances/lifting heavy objects = 1	21.20
		Needing no assistance in the above activities (reference group = 0)	78.80
	Chronic disease status	Without chronic diseases (reference group = 0)	25.52
		Having at least one chronic disease = 1	74.48

As shown in Table 1, overall, 61.94% of the older adults show a younger age identity, and not many of them have access to the Internet (21.14%). In terms of Internet use, the older adults use social functions the most, followed by information acquisition functions, entertainment functions, and life service functions. The older adults have a relatively high level of life satisfaction, and a relatively large proportion of them believe that their physical health is similar to that of their peers. In terms of demographic characteristics, the average age of the older adults in the sample is 71.2 years old. The proportion of male people over 60 is slightly higher than that of female older adults people, the proportion of them of the Han ethnicity is relatively high, the literacy rate of them is relatively high, and the proportion of them living in urban areas is slightly higher. In terms of family characteristics, a relatively high proportion of the older adults are married and have a spouse, a relatively large proportion of them live with their families, and the average number of surviving children is 2.54. In terms of socioeconomic characteristics, the proportion of the people over 60 who are not engaged in paid work (activities), the proportion of them who receive government pension support, and the proportion of them who own real estate are relatively high. In terms of physical health, the number of people over 60 with limitations in instrumental activities of daily living is higher than that of those with limitations in activities of daily living, and most of them suffer from chronic diseases.

The data in Table 2 shows that there are significant urban–rural differences in the older adult’s access to the Internet and their use of Internet functions. The frequency of the older adults living in rural areas accessing the Internet and using various functions is far lower than that of the older adults living in urban areas. Among them, the urban–rural difference in the use of life service functions is the largest. In the longitudinal comparison, it can be seen that, relatively speaking, the older adults living in rural areas use the entertainment and information functions of the Internet more frequently, while the older adults living in urban areas use the life service and social functions of the Internet more often.

**Table 2:** Analysis of urban–rural differences in Internet use (unit: %).

Category name	Urban	Rural	Chi-square value	*p*-value
Use Internet	81.67	18.33	402.2347	0.000
Use of social functions	84.48	15.52	452.0552	0.000
Use of entertainment functions	77.73	22.27	130.3495	0.000
Use of information functions	81.10	18.90	230.7865	0.000
Use of life service	88.27	11.73	131.3734	0.000

## 4 Methods and result analysis

### 4.1 Statistical methods

The theme of this paper’s research is the impact of Internet use on the age identity of the older adults. Considering that the dependent variable is a scale-type variable, a linear regression model is adopted for empirical analysis:


$$\mathrm{Ag}e_{i}=\partial +\text{λInterne}t_{i}+\beta X_{i}+\varepsilon_{i}$$

In the formula, $\mathrm{Ag}e_{i}\;$ represents the age identity variable, $\text{Interne}t_{i}$ represents the core independent variable of Internet use, $\;\varepsilon_{i}\;$ are the control variables, and [random error term] is the random error term. Considering that the above model may have endogeneity problems caused by various objective factors such as reverse causality and omitted variables, which may lead to biased and inconsistent results, the Propensity-Score-Matching method is used in this study to conduct a robustness test on the impact results of Internet use. First, based on the binomial Logistic model, the propensity scores of the older adults Internet use are estimated, and its expression is as follows:


$$E(\text{Interne}t_{i})=P(\text{Interne}t_{i}=1|X_{i}=x_{i})=\frac{1}{1+e^{-x_{i}\beta_{i}}}$$

In the formula, $\text{Interne}t_{i}=1\;$ represents that the older adults use the Internet, $X_{i}$ represents that the older adults do not use the Internet; $\beta_{i}$ is the parameter vector; the propensity score $E(\text{Interne}t_{i})$ is a probability value that simplifies the multi-dimensional covariables into a one-dimensional value. The study mainly focuses on the Average Treatment Effect on the Treated (ATT) of the control group, that is, to verify the impact of Internet use on the age identity of the older adults. The formula is expressed as:


$$\mathrm{ATT}=\frac{1}{N_{t}}\sum m:D_{m}=1[Y_{m}-\sum \mathrm{nQn}(m)w=(m,n)Y_{n}]$$

In the formula, $Y_{m}$ represents the observation value of the Internet use status of the *m*-th older adults person in the control group, $Y_{n}$ represents the observation value of the Internet use status of the *m*-th older adults person in the control group; w=(m,n) is the weight function, and its value range is 0<w=(m,n)≤1 _$w = (*m*,*n*)$_; represents the set of control group individuals matched with the individual *m* in the control group.

Finally, the bias-corrected non-parametric percentile Bootstrap method is used to sample 5,000 times for the mediation effect test, to, respectively, test the mediating effects and influence paths of life satisfaction and self-assessed health compared with peers in the relationship between Internet use and the age identity of the older adults.

### 4.2 Regression analysis

Table 3 shows the regression results of the elder’s Internet use and age identity. All models have passed the Homelessness goodness-of-fit test and the VIF multicollinearity test. The dependent variables of Model 1 to Model 6 are all the age identity of the older adults. According to the regression results of Model 1, the older adults who use the Internet have a relatively significant tendency toward a younger age identity. At the same time, life satisfaction and the health evaluation compared with peers are also significantly related to the age identity of the older adults. Among these three factors, the impact coefficient of Internet use is the highest. Models 2 to 4 conduct a grouped discussion of the older adults according to their actual ages. According to the regression results, the impact of the Internet on the age identity of the older adults is more reflected in the younger age group (60–69 years old). A more youthful age identity is observed among older people Internet users compared to non-users. However, the role of Internet use in the age identity of the 70–79 age group is minimal, and it has no significant effect on the age identity of the older adults in the age group of 80 years old and above. Models 5 to 6 conduct a grouped analysis of the older adults population according to the type of residence. According to the regression results, the impact of Internet use on the younger age identity of the older adults has played a positive role both in urban and rural areas. In cities, the impact coefficient of Internet use is 1.722, and in rural areas, the impact coefficient of Internet use is 1.651. Hypothesis 1 is partially verified.

**Table 3:** Regression results of the Internet use by the older adults and their age identity.

Independent variable	Model 1	Model 2	Model 3	Model 4	Model 5	Model 6	Model 7
	Full sample	60–69岁	70–79岁	80岁及以上	Urban	Rural	Internet use
Internet use	1.615*** (0.202)	1.975*** (0.237)	0.765* (0.447)	1.747 (1.354)	1.722*** (0.238)	1.651*** (0.45)	
Life satisfaction	0.273*** (0.103)	0.476*** (0.125)	0.103 (0.166)	−0.114 (0.365)	0.643*** (0.142)	−0.192 (0.15)	
Relative health	0.536*** (0.129)	0.839*** (0.163)	0.277 (0.211)	0.527 (0.405)	0.777*** (0.178)	0.22 (0.185)	
Age	0.453*** (0.017)	0.388*** (0.041)	0.561*** (0.05)	0.669*** (0.092)	0.506*** (0.023)	0.372*** (0.026)	−0.135*** (0.007)
Female	0.249 (0.171)	0.14 (0.209)	0.221 (0.279)	0.614 (0.622)	0.199 (0.221)	0.378 (0.272)	0.036 (0.066)
Han ethnicity	1.074*** (0.306)	−0.126 (0.391)	1.178*** (0.45)	3.989*** (1.005)	1.144** (0.525)	0.806** (0.38)	0.376** (0.162)
Married with a spouse	0.46** (0.228)	0.126 (0.305)	0.915*** (0.338)	0.184 (0.654)	0.3 (0.302)	0.635* (0.342)	0.3*** (0.097)
Literate	0.208 (0.211)	0.382 (0.271)	−0.58* (0.315)	1.068 (0.651)	0.356 (0.322)	0.051 (0.286)	1.163*** (0.122)
Living alone	0.276 (0.325)	−0.13 (0.486)	0.576 (0.452)	0.463 (0.796)	0.061 (0.434)	0.532 (0.49)	−0.132 (0.149)
Rural	−0.192 (0.213)	0.218 (0.276)	−0.539 (0.334)	0.29 (0.729)			−1.054*** (0.089)
Number of surviving children	0.018 (0.08)	−0.081 (0.109)	0.106 (0.115)	0.084 (0.188)	−0.009 (0.115)	0.021 (0.108)	−0.22*** (0.037)
Employed	0.249 (0.209)	−0.097 (0.258)	0.255 (0.348)	0.132 (0.865)	0.874** (0.372)	−0.284 (0.254)	0.397*** (0.089)
With pension	0.467 (0.324)	0.968** (0.409)	0.224 (0.521)	−1.861 (1.381)	0.74 (0.484)	0.411 (0.432)	−0.01 (0.12)
Annual personal income	0.332*** (0.073)	−0.142 (0.096)	0.286*** (0.11)	1.5*** (0.225)	0.456*** (0.096)	0.139 (0.112)	0.267*** (0.027)
With real estate	0.206 (0.409)	−0.44 (0.573)	0.562 (0.599)	−0.01 (0.941)	−1.118* (0.648)	1.018** (0.513)	0.974*** (0.278)
ADL disability	−0.852*** (0.297)	−0.634 (0.423)	−0.491 (0.41)	−1.286 (0.798)	−0.816** (0.384)	−0.91** (0.46)	−0.006 (0.122)
IADL disability	−0.05 (0.236)	−0.538 (0.331)	0.35 (0.326)	−0.743 (0.673)	−0.295 (0.303)	0.147 (0.376)	−0.203** (0.099)
With chronic disease	−1.179*** (0.2)	−0.013 (0.234)	−1.981*** (0.346)	−3.717*** (0.745)	−0.779*** (0.267)	−1.608*** (0.302)	0.414*** (0.076)
_cons	−35.007*** (1.523)	−27.214*** (2.969)	−41.333*** (3.865)	−60.702*** (8.067)	−40.723*** (1.991)	−26.148*** (2.278)	
/cut1							−3.657*** (0.625)
Observations	7,665	3,839	2,653	1,173	4,598	3,067	7,665
Pseudo *R*^2^	0.168	0.061	0.084	0.154	0.198	0.132	0.233

Standard errors are in parentheses ***p < 0.01, **p < 0.05, *p < 0.1.

### 4.3 Robustness test

The binomial Logistic model was employed to analyze the influencing factors of whether the older adults use the Internet. The regression results are shown in Model 7. It can be seen that among the independent variables, being Han Chinese, being literate, being married with a spouse, being employed, having an annual personal income, owning a property, and suffering from chronic diseases are all positively correlated with the Internet use of the older adults. The actual age of the older adults, the number of surviving children, and ADL (Activities of Daily Living) disability are negatively correlated with Internet use. It is worth noting that the older adults living in rural areas have a lower rate of Internet access compared with those living in urban areas.

In this study, the one-to-one nearest neighbor matching method, kernel matching method, and radius matching method were, respectively, used to test the impact of Internet use on the elder’s age identity, life satisfaction, and health comparison with peers. Before the test, the parallelism assumption of the matching was verified. Judging from the test results of the balance assumption, after matching, except for the variables that were already insignificant in individual models, there were no significant differences in other variables between groups (*p* > 0.05), indicating that the matching has well balanced the differences between the control group and the treatment group in the sample, and the balance assumption is satisfied.

Table 4 reports the regression results of the average treatment effect. Under the three test methods, the regression coefficient of the average treatment effect is significantly positive. The results indicate that Internet use has a significant positive impact on older adults’ tendency toward a younger age identity, their life satisfaction, and their health comparisons with peers. These regression findings are robust.

**Table 4:** Results of the average treatment effect.

Independent variable			Using the Internet	Not using the Internet	Average treatment effect (ATT)	Standard error	*T*-value
Nearest neighbor matching within the caliper	Age identity	Before matching	3.278	3.605	1.790	0.235	7.61***
		After matching	3.330	1.540			
	Life satisfaction	Before matching	2.926	2.738	0.136	0.028	4.84***
		After matching	2.906	2.770			
	Relative health	Before matching	2.296	2.102	0.089	0.026	3.36***
		After matching	2.267	2.178			
Radius matching	Age identity	Before matching	3.278	3.605	2.041	0.244	8.38***
		After matching	3.275	1.234			
	Life satisfaction	Before matching	2.926	2.738	0.148	0.028	5.22***
		After matching	2.925	2.780			
	Relative health	Before matching	2.296	2.102	0.115	0.026	4.41***
		After matching	2.296	2.181			
Kernel matching	Age identity	Before matching	3.278	3.605	2.053	0.244	8.42***
		After matching	3.275	1.222			
	Life satisfaction	Before matching	2.926	2.738	0.148	0.028	5.20***
		After matching	2.925	2.777			
	Relative health	Before matching	2.296	2.102	0.115	0.026	4.38***
		After matching	2.296	2.181			

***p < 0.01, **p < 0.05, *p < 0.1.

To address the potential reverse causality between Internet use and age identity (i.e., that a younger self-perception may increase Internet adoption), this study employs an instrumental variable (IV) approach. We select the availability of an Internet signal in the respondent’s residence as the IV and estimate the relationship using a two-stage least squares (2SLS) method. This choice satisfies the core conditions for a valid instrument: it is correlated with individual Internet use (relevance) but is unlikely to be directly influenced by, or directly influence, an older adult’s age identity (exogeneity). The results of the instrumental variable (IV) regression, reported in Table 5, address potential endogeneity concerns. The first-stage regression demonstrates that the instrument is a powerful predictor of Internet usage, with a highly significant coefficient (*p* < 0.001). The accompanying *F*-statistic of 202.71 decisively rejects the null hypothesis of weak instruments, providing confidence in the identification strategy. Proceeding to the second stage, the estimate for Internet usage remains positive and statistically significant at the 0.1% level. This consistency underscores the robustness of the baseline finding that Internet usage promotes a younger age identity.

**Table 5:** Instrumental variable regression.

Independent variable	Phase l regression	Phase II regression
Instrumental variable	0.373*** (0.009)	
Internet Use		1.563*** (0.497)
Other variables have been controlled		
_cons	0.697*** (1.578)	−32.987*** (0.057)
Observations	7,655	7,655
R-squared	0.346	0.164
Phase 1-value	202.71	
Kleibergen–Paaprk Wald *F* statistic	1757.572 [16.38]	

Standard errors are in parentheses ***p < 0.01, **p < 0.05, *p < 0.1. The critical value of the weak instrument identification *F*-test at the 10% significance level.

A sensitivity analysis was conducted using a categorical regression model. The sample was divided into three groups based on whether their subjective age was younger than, equal to, or older than their chronological age. As shown in Table 6, Internet use significantly promoted a younger age identity in the “younger subjective age” group (*p* < 0.05, Model 1). A positive effect was also observed at a marginal significance level in the “equal subjective age” group (*p* < 0.1, Model 2). However, the relationship was non-significant in the “older subjective age” group. These findings collectively validate the robustness of the main results.

**Table 6:** Classification regression.

Independent variable	Model 1 subjective age < actual age	Model 2 subjective age = actual age	Model 3 subjective age > actual age
Internet use	1.975*** (0.237)	0.765* (0.447)	1.747 (1.354)
Life satisfaction	0.476*** (0.125)	0.103 (0.166)	−0.114 (0.365)
Relative health	0.839*** (0.163)	0.277 (0.211)	0.527 (0.405)
Other variables have been controlled			
_cons	−27.214*** (2.969)	−41.333*** (3.865)	−60.702*** (8.067)
Observations	3,839	2,653	1,173
R-squared	0.061	0.084	0.154

Standard errors are in parentheses ***p < 0.01, **p < 0.05, *p < 0.1.

### 4.4 Analysis of the mediating effect

To further clarify the influence mechanism of Internet use on the age identity of the older adults, it can be seen from Model 1 and Table 7 that Internet use has a significant impact on the age identity, life satisfaction, and health comparison with peers of the older adults. Judging from the influence coefficients in Model 1, Internet use was identified as the most influential predictor of a younger subjective age among the older adults, followed by health comparison with peers, with life satisfaction ranking last.

**Table 7:** Analysis of the mediating effect.

Independent variable	Model 8	Model 9	Model 10
	Age identity	Life satisfaction	Relative health
Use the Internet	1.726*** (0.204)	0.118*** (0.024)	0.089*** (0.022)
Life satisfaction			0.24*** (0.011)
Relative health		0.312*** (0.014)	
Other variables have been controlled			
_cons	−33.122*** (1.503)	1.745*** (0.152)	1.68*** (0.134)
Observations	7,665	7,665	7,665
R-squared	0.164	0.108	0.156

Standard errors are in parentheses ***p < 0.01, **p < 0.05, *p < 0.1.

The Process3.0 plugin of SPSS was used, and Model 4 was selected to verify the mediating effects of life satisfaction and health comparison with peers on the relationship between Internet use and the age identity of the older adults. The results are shown in Table 8. In the model of the impact of Internet use on the age identity of the older adults, the mediating effect of life satisfaction is [0.011, 0.081], and the mediating effect of health comparison with peers is [0.034, 0.120]. Since the intervals do not include 0, it indicates that both variables play a mediating role. The direct effect of Internet use on the age identity of the older adults is significant (*p* < 0.001). Therefore, life satisfaction and health comparison with peers play a partial mediating role. Hypothesis 2 is verified. Using the Internet can not only have a direct positive impact on age identity, but also make the older adults present a more youthful age identity by improving their life satisfaction and scores of health comparison with peers.

**Table 8:** Bootstrap test of the mediating effect.

Independent variable		Mediating variable	*B*	SE	LLCI	ULCI	*p*
Internet access	Direct effect		1.615	0.218	1.189	2.041	0.000
	Mediating effect	Life satisfaction	0.043	0.018	0.012	0.084	
		Relative health	0.068	0.021	0.034	0.115	

### 4.5 Analysis of the mechanism of action

To further explore the mechanism underlying the impact of Internet use on older adults’ age identity, this study focuses on examining how Internet functions and usage behaviors influence their age identity. As shown in Table 9, Model 11 shows that the older adults who use the Internet for socializing, entertainment, and obtaining life services all show a more significant youthful-oriented age identity. Model 12 shows that the older adults who use the Internet to obtain information and life-related services have higher life satisfaction (*p* < 0.005), while the social and entertainment functions of the Internet are negatively correlated with life satisfaction (*p* < 0.1). Model 13 shows that the social function of the Internet is significantly positively correlated with the elder’s health comparison with peers (*p* < 0.005), and the life-service function is weakly positively correlated with it (*p* < 0.1). Model 14 shows that both health comparison with peers and life satisfaction are significantly correlated with the age identity of the older adults. Therefore, a further analysis of the mediating effect is carried out.

**Table 9:** Internet use functions and the age identity of the older adults.

Independent variable	Model 11	Model 12	Model 13	Model 14
	Age identity	Life satisfaction	Relative health	Age identity
Using the social function of the Internet	1.198*** (0.302)	−0.062* (0.037)	0.118*** (0.034)	1.147*** (0.301)
Using the entertainment function of the Internet	0.559* (0.3)	−0.064* (0.039)	−0.029 (0.036)	0.607** (0.296)
Using the information function of the Internet	0.017 (0.317)	0.213*** (0.04)	0.002 (0.037)	−0.078 (0.315)
Using the life-service function of the Internet	1.03*** (0.368)	0.126*** (0.042)	0.07* (0.041)	0.928** (0.365)
Health comparison with peers		0.311*** (0.014)		0.523*** (0.129)
Life satisfaction			0.24*** (0.011)	0.285*** (0.104)
Other variables have been controlled				
_cons	−33.13*** (1.504)	1.778*** (0.152)	1.662*** (0.134)	−35.017*** (1.524)
Observations	7,665	7,665	7,665	7,665
R-squared	0.164	0.111	0.157	0.168

Standard errors are in parentheses ***p < 0.01, **p < 0.05, *p < 0.1.

Table 10 shows the test of the mediating effect of life satisfaction and health comparison with peers on the relationship between Internet functions and the youthful age identity of the older adults. After adding the mediating variables, the direct effect of the Internet’s social function on the more youthful—oriented age identity of the older adults is still significant, but the direct effect becomes smaller (*p* < 0.01). The mediating effect of life satisfaction is [−0.039, 0.012], and the interval contains 0, so the mediating effect is not significant. The mediating effect of health comparison with peers is [0.021, 0.116], and the interval does not contain 0, indicating that health comparison with peers plays a mediating role between the Internet’s social function and age identity.

**Table 10:** Bootstrap test of the mediating effect of Internet use functions.

Independent variable		Mediating variable	*B*	SE	LLCI	ULCI	*p*
Social function	Direct effect		1.147	0.328	0.504	1.791	0.001
	Mediating effect	Life satisfaction	−0.008	0.012	−0.039	0.012	
		Relative health	0.059	0.024	0.021	0.116	
Entertainment function	Direct effect		0.607	0.347	−0.074	1.288	0.081
	Mediating effect	Life satisfaction	−0.023	0.015	−0.063	−0.001	
		Relative health	−0.025	0.021	−0.073	0.012	
Information function	Direct effect		−0.078	0.356	−0.775	0.619	0.826
	Mediating effect	Life satisfaction	0.066	0.027	0.021	0.127	
		Relative health	0.030	0.023	−0.006	0.085	
Life-service function	Direct effect		0.928	0.421	0.103	1.754	0.028
	Mediating effect	Life satisfaction	0.046	0.022	0.013	0.100	
		Relative health	0.057	0.027	0.014	0.122	

After adding the mediating variables, the direct effect of the Internet’s entertainment function is [−0.074, 1.282], and the interval contains 0, so the direct effect is not significant. The mediating effect of life satisfaction is [−0.063, −0.001], and the interval does not contain 0, so the mediating effect is significant. The mediating effect of health comparison with peers is [−0.073, 0.012], and the interval contains 0, so the mediating effect is not significant. Therefore, it can be seen that life satisfaction plays a complete mediating role in the impact of the Internet’s entertainment function on the age identity of the older adults and reversely masks the impact of the entertainment function on age identity. Hypothesis 3b is not verified.

After adding the mediating variables, the direct effect of the Internet’s information function is [−0.775, 0.619], and the interval contains 0, so the direct effect is not significant. The mediating effect of life satisfaction is [0.021, 0.127], and the interval does not contain 0, so the mediating effect is significant. The mediating effect of health comparison with peers is [−0.006, 0.085], and the interval contains 0, so the mediating effect is not significant. Therefore, it can be seen that life satisfaction plays a complete mediating role in the impact of the Internet’s information function on the age identity of the older adults.

After adding the mediating variables, the direct effect of the Internet’s life-service function is still significant, but the direct effect becomes smaller (*p* < 0.05). The mediating effect of life satisfaction is [0.013, 0.010], and the mediating effect of relative health is [0.014, 0.122]. The intervals do not contain 0, and the mediating effects are both significant. Therefore, it can be seen that both play a partial mediating role. Hypothesis 3a is partially verified.

## 5 Conclusions and discussions

### 5.1 Internet use has a positive impact on the positive aging attitude of the older adults

The impact of Internet use on the age identity of the older adults has played a positive role both in urban and rural areas. However, this impact exhibits age-related differences and is primarily manifested in the young-old group (aged 60–69). As older adults advance in age, the role of the Internet becomes statistically insignificant.This may be attributed to the fact that, compared with older age groups, young-old adults (60–69 years old) still retain a certain level of learning capacity and have a higher likelihood of accessing the Internet. It may also be that as the older adults enter the advanced age stage, their physical health deteriorates, and the happiness incentive effect of the Internet is not obvious (33). In addition, disability, the number of living children, and living in rural areas are also significantly negatively correlated with the Internet usage rate of the older adults, which is consistent with previous studies, that is, the older adults face problems such as age barriers, knowledge barriers, health barriers, and insufficient support from their children when accessing the Internet (34). The Internet usage rate of the older adults in rural areas urgently needs to be improved.

### 5.2 Life satisfaction and health comparison with peers play a partial mediating role in the impact of the Internet on the positive aging attitude of the older adults

Internet use exerts a positive impact on both older adults’ life satisfaction and their health-related social comparison with peers. Specifically, engaging with the Internet helps older adults develop a younger age identity by enhancing their life satisfaction and facilitating more favorable health comparisons with peers. Many domestic studies have shown that the use of the Internet can significantly improve the happiness and life satisfaction of the older adults. The research conclusions of this article are consistent with those of previous studies. Further research has found that there is a positive correlation between Internet use and the health comparison of the older adults with their peers. Self-rated Health is a frequently used health prediction indicator in academic research, which is different from the “objective health status.” It is a comprehensive result of objective health status and subjective health perception and is an effective factor in predicting all-cause mortality (35). Social comparison theory believes that people’s evaluation of themselves stems from comparison with others. Judging from the impact coefficient of the model, health comparison with peers has a more important impact on the younger age identity of the older adults compared with life satisfaction, and has become an important mediating mechanism between the role of the Internet and age identity.

### 5.3 The impact of the uses of the Internet on the positive aging attitude of the older adults has differences

The social function of the Internet has the most significant effect, and health comparison with peers plays a partial mediating role in it. The life service function of the Internet also has a significant effect and plays a role by improving life satisfaction and health comparison with peers. The entertainment function of the Internet ranks third. The entertainment function is negatively correlated with life satisfaction, indicating that life satisfaction inversely obscures the impact of the entertainment function on age identity. The information function of the Internet has no significant effect, and life satisfaction plays a complete mediating role in the impact of the information function on the age identity of the older adults. In addition, it is worth noting that the social function of the Internet is positively correlated with the health comparison of the older adults with their peers but negatively correlated with life satisfaction. One possible reason is that the Internet enhances the social connection of the older adults, making it easier for them to compare their health with their peer group. However, in the minds of the older adults, interacting with family and friends through the screen still cannot compare with the emotional comfort brought by face-to-face communication. The entertainment function of the Internet is also negatively correlated with life satisfaction. One possible reason is that for the older adults with weak self-control, excessive use of the Internet may reduce their sleep time (36), make them feel more lonely (18), and indulging in the entertainment function of the Internet will reduce the interaction between the older adults and those around them. In contrast, the Internet’s information function exerts a positive impact on older adults’ life satisfaction, while its life service function plays a proactive role in boosting three key aspects of their well-being: a younger self-perceived age, life satisfaction, and positive health comparisons with peers. From the perspective of empowerment, the older adults who want to live a good old age need to empower themselves to make up for the lack of objective and relative power caused by aging and the changes of modern society (37). This article believes that the life service function of the Internet can provide more convenience for the older adults in health management, shopping, transportation, learning and training, and financial management, endow the older adults with more independent action ability, stimulate their learning ability, strengthen their independence, and improve their ability to master their own lives and achieve self-development. Therefore, it should become the main development direction of empowering the older adults for positive aging through the Internet.

Based on the above conclusions, this article suggests that in the practice of the positive aging strategy, the following should be considered:

First, increase the opportunities for the older adults to access the Internet and narrow the “digital divide” between urban and rural areas. The research shows that there are significant urban–rural differences in the Internet access rate of the older adults and the usage rate of various Internet functions. The popularity of the Internet and the role of life service functions in rural areas are far less than those in urban areas. Therefore, it is imperative to address the urban-rural gap in Internet infrastructure development, lower the barriers to Internet access for older adults, and encourage enterprises to conduct aging-friendly renovations of Internet products. Additionally, efforts should be made to provide targeted guidance on Internet usage, enhance older adults’ digital literacy, and motivate them to make greater use of the Internet’s life service functions—such as learning and health management—to reap the tangible benefits of digitalization in their daily lives.

Second, based on the needs of the older adults, extend the functions of smart older adults care services. The research shows that the social service functions of the Internet have a positive promoting effect on the younger age identity, life satisfaction, and health comparison with peers of the older adults. However, disabled older adults and those residing in rural areas still have limited access to the Internet and its life service functions. Therefore, we should advance the integration of “production, education, research, application, and management,” and construct an empowerment-centered smart older adults care service system. By targeting the heterogeneous needs and capabilities of different older adults groups, we should, in particular, provide more intelligent life support for disabled older adults, connect additional older adults care service resources for rural-dwelling older adults, and extend the dividends of the Internet to disabled and older adults populations across urban and rural areas.

Third, encourage inter-generational digital feedback and offline interaction. The research shows that the number of living children is negatively correlated with the Internet usage rate of the older adults. In view of the positive significance of Internet use for the empowerment and happy aging of the older adults, this article believes that children should provide hardware devices for the digital integration of the older adults and teach more Internet usage knowledge to them. As the most direct and effective inter-generational technical and emotional support, digital feedback should be valued by family members. Young volunteers in the community can also play a positive role in it, aiming to activate and improve the learning ability of the older adults, improve their ability to distinguish between bad network information, and help them share the achievements of social development through digital integration. In the context of a mobile society, the use of the Internet can, to a certain extent, strengthen inter-generational communication and reduce the social barriers of the older adults. However, it should be noted that online communication cannot completely replace the important significance of offline interaction for the spiritual comfort of the older adults.

In summary, this research contributes a nuanced understanding of Internet use by examining the distinct pathways through which specific functions-social, entertainment, information, and life services-influence older adults. The analysis reveals that these functions operate via unique mediating mechanisms (life satisfaction and peer health comparison), among which life services emerge as the most impactful. Consequently, this study clarifies the mechanisms of Internet use, thereby yielding valuable insights for designing targeted digital interventions. This study has three main limitations. First, operationalizing age identity as the difference between chronological and subjective age, though practical, is a simplified measure that may not capture the construct’s full complexity. Second, the cross-sectional data prevent causal conclusions about the relationship between Internet use and age identity. Third, the use of self-reported data, including a simplistic dichotomization of Internet use, introduces potential for bias and overlooks nuances in digital engagement. To advance this line of inquiry, future studies would be well-advised to incorporate multi-dimensional scales for a comprehensive assessment of age identity, while also utilizing longitudinal or experimental designs to robustly ascertain causality.

## Data Availability

The datasets presented in this study can be found in online repositories. The names of the repository/repositories and accession number(s) can be found at: http://class.ruc.edu.cn/.
